# Clinical-Radiological Correlation of Retained Silicone Sponge Presenting as Orbital Inflammation

**DOI:** 10.1155/2016/5291587

**Published:** 2016-06-16

**Authors:** Tal J. Rubinstein, John Clemett, Charles D. Birnbach, Steven J. LauKaitis, Bryan S. Sires

**Affiliations:** ^1^Allure Laser and Medispa/Aesthetic Eye Associates, 625 4th Avenue, Suite 302, Kirkland, WA 98033, USA; ^2^Overlake Hospital Medical Center, Department of Radiology, 1035 116th Avenue NE, Bellevue, WA 98004, USA; ^3^Vitreoretinal Associates, 1750 112th Avenue NE No. D050, Bellevue, WA 98004, USA

## Abstract

A 32-year-old female who underwent scleral buckle removal presented 5 weeks postoperatively with a red, fluctuant subconjunctival mass. CT scan identified an irregularly bordered, hypoattenuated lesion next to the globe with the density of air. Ophthalmic plastic and reconstructive surgeons were consulted to evaluate orbital cellulitis with intraorbital gas, at which point it was deemed that the hypoattenuated mass was likely a retained sponge element based on its radiological features. Additional surgical exploration identified the retained silicone sponge. This clinical photographic-radiological correlation of retained silicone sponges presenting as orbital inflammation reminds surgeons to meticulously explant buckle material.

## 1. Introduction

Scleral buckle infections range in clinical appearance from pain, conjunctivitis, and redness to orbital cellulitis or even scleral abscesses with vitritis [1–4]. Treatment of such infections includes systemic and topical antibiotics at first but often requires explanation of the buckle [1–3, 5]. Scleral buckle elements may also cause sterile inflammation or orbital pseudocellulitis [3, 6, 7].

The radiologic features of scleral buckles and infected scleral buckles with or without orbital cellulitis have been described [1–3, 8]. The prior cases and case series that cite specific clinical signs and symptoms with radiographic correlations describe orbital cellulitis in primary scleral buckles, but not in eyes with retained buckles after prior explantation. This is a case of a patient with signs of orbital inflammation five weeks after scleral buckle explantation due to a retained silicone sponge fragment. Based on Medline literature review, this is a unique case of a clinical photographic-radiologic correlation of a retained silicone sponge causing such signs.

## 2. Case

A 32-year-old female presented to the emergency room with a red, painful right eye. She had undergone multiple retinal detachments in that eye treated with scleral buckling, last of which was performed in Russia five years priorly. No international medical records were available. Five weeks prior to presentation, in the United States, she underwent explanation of an exposed and presumed infected scleral buckle with discharge and discomfort, in which sponge material was removed from the temporal quadrant. The cultures of the sponge were negative. In the emergency room a CT scan of the orbits was obtained. The official radiology read of the CT identified a lobulated air or gas collection lateral to the globe with mass effect on the globe and adjacent intermediate soft tissue attenuation.

**Figure 1:**
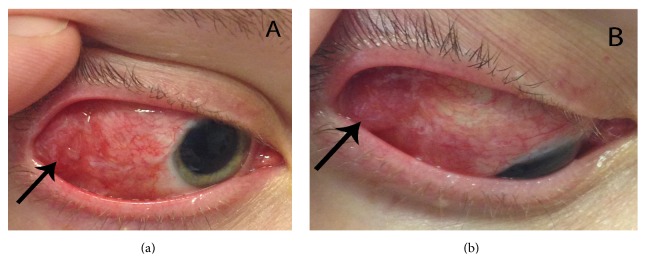
(a) Clinical photograph of the patient's right eye. Note the red, elevated mass temporally underneath a closed and scarred conjunctiva. Pupil is pharmacologically dilated. (b) Right eye in downgaze and adduction.

The patient was referred to ophthalmic plastic and reconstructive surgeons for the concern for intraorbital air or gas from an infectious cause. She was well-appearing and afebrile. She had 20/40 vision OD with correction. Intraocular pressure was 19. She had no APD and normal color vision. She had −1 abduction OD. The right eye exhibited 1 mm of relative exophthalmos. She had no periorbital edema. Temporally and superotemporally, she had a fluctuant, painful, elevated subconjunctival mass (Figures 1(a) and 1(b)). The conjunctiva was scarred and closed temporally. The rest of the ocular exam was normal other than retinal laser scars.

**Figure 2:**
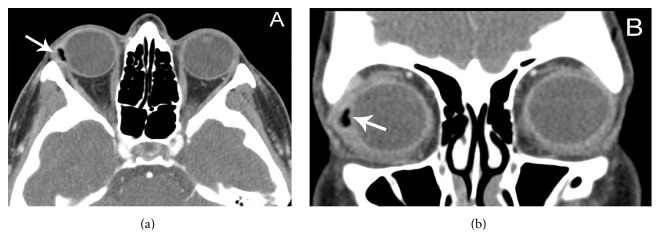
(a) Axial CT image identifying a hypoattenuated lesion with slight localized mass effect on the globe with surrounding soft tissue attenuation. Note the irregular, jagged borders of the lesion. (b) Coronal cut shows more definitive surrounding soft tissue attenuation. The hypoattenuation mass, which was confirmed by surgical explantation to be a retained silicone sponge element, was noted to have an attenuation of −284 Hounsfield units.

On review of the CT scan, in light of the history and clinical findings, it was presumed that the “air” was likely a retained silicone sponge, which correlated in location with the subconjunctival mass (Figures 2(a) and 2(b)). The sponge element had an irregular, jagged border radiographically. Surgical exploration identified a retained silicone sponge element. Oral antibiotics were started. Cultures were negative. The patient's symptoms and clinical exam normalized after surgery.

## 3. Discussion

Scleral buckle-associated infection or inflammation may present with a range of signs and symptoms, from pain, redness, foreign body sensation, and discharge to fulminant orbital cellulitis with vision loss, proptosis, chemosis, and ophthalmoplegia [1–3, 5, 6, 9, 10]. Considering the overall mild set of signs and symptoms, the clinical presentation of this patient may be due to inflammatory rather than infectious causes, but distinguishing infectious versus noninfectious inflammation may be difficult. On CT imaging, infected buckles are often associated with preseptal edema, diffuse scleral thickening, or extraocular muscle thickening, which this patient's imaging lacked [1]. The adjacent soft tissue attenuation observed on this patient's CT is likely related to the inflammation affecting nearby tissues. The culture of the retained sponge fragment was negative, but buckle cultures are often negative in nonexposed buckles and may have been negative due to antibiotic therapy [5]. Atypical organisms such as mycobacteria or fungi may also display negative cultures in standard bacterial cultures. Both infectious and noninfectious inflammatory lesions due to retained scleral buckle elements should be approached by exploration in order to remove the residual foreign body.

Silicone buckles form a foreign body encapsulation, which may make dissection difficult, leading to a higher chance of incomplete removal with retention of some of the material [11]. A high index of suspicion for retained buckle material is therefore necessary in patients with new or continued signs or symptoms of orbital inflammation or cellulitis after initial buckle removal.

Scleral buckle elements have characteristic CT findings. Solid silicone bands appear hyperdense on CT, but silicone sponges appear hypodense [2, 8, 12]. The density of the sponge is similar to air because of the air pockets within the material, with an attenuation of −250 to −400 Hounsfield units [8]. The borders of the sponges are typically smooth on CT [1, 2, 8, 12]. Infected sponges are more highly attenuated [1]. The differential diagnosis of a hypoattenuated mass of the orbit on CT imaging includes air, fat, dacryocystitis, abscess, mucocele, or cyst [13]. Most of these conditions, however, would not give an attenuation as low as seen with air. Importantly, the irregular and jagged borders of the silicone buckle on the CT scan, possibly due to prior surgical explantation and manipulation, make the radiological diagnosis of a silicone sponge more difficult. To the author's knowledge, such a radiological appearance is unique to the literature.

Sharma et al. published a clinical-radiographic correlation of infected scleral buckles [3]. Two of the patients had persistent symptoms after initial scleral buckle explantation with subsequent CT identifying retained buckle materials, like this patient. However, three important features make this case distinguishable and unique. First, those patients displayed persistent conjunctivitis rather than an inflammatory orbital mass, as in this case. Second, this case includes photographic correlation of the mass and its location to the CT finding, which is unique in the literature. Finally, the buckle materials in those cases were solid silicone bands and hyperdense on CT, rather than the hypodense sponge material in this case.

This case has important implications to clinical practice. In cases of orbital inflammation after scleral buckle explantation, orbital imaging may demonstrate a retained buckle element located at the site of the inflammation. The possibility for retained buckle material should be considered especially in patients with scarred orbital planes from prior surgeries, as scarring can make removal of the complete fragments difficult. Furthermore, in consultation for orbital inflammation or cellulitis, the eye care provider must be familiar with the CT imaging characteristics of retained scleral buckle elements. Specifically, retained silicone sponge elements are hypoattenuated and small and may have irregular borders, so they may be misinterpreted for air or gas from an infectious cause. Failure to correctly identify the retained elements can delay appropriate surgical care.
